# *Brugia malayi* filarial helminth-derived extracellular vesicles suppress antigen presenting cell function and antigen-specific CD4+ T cell responses

**DOI:** 10.3389/fimmu.2024.1436818

**Published:** 2024-10-07

**Authors:** Gayatri Sanku, Alessandra Ricciardi, Neelam R. Redekar, Paul Schaughency, Justin Lack, Pedro H. Gazzinelli-Guimaraes, Thomas B. Nutman

**Affiliations:** ^1^ Laboratory of Parasitic Diseases, National Institute of Allergy and Infectious Diseases, National Institutes of Health (NIH), Bethesda, MD, United States; ^2^ Integrated Data Science Section (IDSS), National Institute of Allergy and Infectious Diseases (NIAID), National Institutes of Health (NIH), Bethesda, MD, United States

**Keywords:** *Brugia malayi*, extracellular vesicle (EV), microfilariae excretory-secretory protein, T cell, helminth

## Abstract

**Introduction:**

Live microfilariae (mf) and mf-derived extracellular vesicles (EVs) have been shown to modulate human antigen presenting cell (APC) function, most notably by suppressing the induction of IL-12 (and other pro-inflammatory cytokines) following activation with LPS and interferon-y.

**Methods:**

To explore further how EVs alter human APC function, we studied the effect of mf and EVs on human elutriated monocyte-derived dendritic cells (DC) following exposure to Mf, mf-derived excretory/secretory (E/S) products, E/S depleted of EVs through ultracentrifugation and purified EVs.  After demonstrating that the measurable responses induced by live mf could be recapitulated by EVs and EV-containing E/S, we next performed RNAseq analysis of human DC following exposure to live mf, EVs, E/S, or EV-depleted E/S.

**Results:**

In our analyses of the data for the DC, using a false discovery rate (FDR)<0.05, EV-exposed DC had induced the expression of 212 differentially expressed genes (DEGs) when compared to unexposed DC and 157 when compared to E/S-depleted EVs.  These genes were enriched in GO biological processes associated with neutrophil degranulation and 15 DEGs associated with KEGG Lysosome pathways. IPA analysis point to immune dysregulation. We next aimed to understand the intracellular processes altered by EVs and the effect these have on effector T cells. When SARS CoV-2 Membrane-specific CD4+ TCLs were assessed following EV conditioning of autologous DC and activation with the SARS CoV-2-Membrane peptide pool, we found conditioning reduced the frequency of SARS CoV-2 Membrane-specific CD3+ CD4+ CD154+ cells (p=.015). Similarly, EV-conditioning of SARS CoV-2 Membrane-specific CD3+ CD4+ cells induced fewer cell capable of producing IFN-γ (p=.045).

**Discussion:**

Taken together, our data suggest a modulatory role of EVs on APC function that likely leads to defects in T cell effector function.

## 1 Introduction

Among the various filarial infections that together infect >200 million people worldwide, those with lymphatic filariasis, caused by *Brugia* spp. and *W. bancrofti* infection, are the most severely affected. A common immunologic feature of all the filarial infections (and most notably those that are bloodborne) is the expansion of parasite antigen-specific Th2 cells well as the expansion of IL-10-producing CD4+ T cells (1), processes largely associated with the presence of circulating microfilariae (Mf), the parasite stage that provides the most significant source of excreted/secreted (E/S) parasite antigen. Additionally, parasite antigen-specific Th1 responses are muted leading to what has been termed antigen-specific T cell hypo-responsiveness; this suppressed parasite-specific memory response spills over to non-parasite bystander antigens with persistent, longstanding infection (2, 3).

A number of hypotheses have been put forth to explain the T cell alterations seen in chronic filarial infections including: 1) TGF-β receptor engagement (4); 2) induction of Tregs (5); and 3) APC dysfunction (6), mostly triggered by E/S products. Previous studies have shown that E/S antigens can drive CD14+ monocytes to become capable of modulating both T cell and lymphatic endothelial functions (7). Moreover, E/S products have been shown to modulate dendritic cell (DC) maturation, leading to altered immune responses (8). Studies have demonstrated that exposure of DC to helminth E/S products can inhibit the upregulation of costimulatory molecules, such as CD80 and CD86 and promote the downregulation of antigen uptake receptors, such as DEC-205 and mannose receptor, thereby preventing full maturation of DC (9). This immature DC phenotype is associated with the induction of regulatory T cell (Treg) responses (10) which may contribute to immune suppression and parasite persistence. Filarial helminth infections have also been shown to interfere with the Toll-like receptor (TLR) signaling pathway in DC, again resulting in impaired DC maturation and cytokine production (11).

Because extracellular vesicles (EVs) are part of E/S products (12–14), it has been suggested that EVs can interact with a range of host cells and may be mediators or central drivers of immune modulation. These interactions could suppress APC functions and alter the generation of antigen-specific T cells (15). Interestingly, EVs produced by intestinal nematodes have also been associated with immunoregulation (12). *Heligmosomoides polygyru*s-derived EVs can suppress the activation of type 2 innate lymphoid cells (ILC2s) (16) by inhibiting the expression of IL-33 receptor, which is essential for the activation of ILC2s.

Notwithstanding, Mf-derived EVs, known to be released in large quantities and being capable of modifying intracellular functions and cellular signaling in APCs (12), have previously been shown to alter some intracellular processes including mTOR signaling (17). Thus, EVs have emerged as attractive carriers of proteins, lipids, and nucleic acids that may penetrate host defensive barriers and increase cellular susceptibility to immune suppression (18). Having previously shown that these Mf-derived EVs are heterogenous in size with both exosome and microvesicle-like particles that contain protein cargo with mammalian exosome markers (elongation factor 1- α, histones, heat shock proteins and ATP synthase) (17), we have previously demonstrated EVs to be readily internalized within human monocytes, where they affect key intracellular functions (17). However, the underlying mechanisms on how Mf-derived EVs alter the molecular program of APCs and its subsequent effect on antigen-specific T cell effector responses are less studied.

Therefore, we sought to understand the role played by these Mf-derived EVs in modulating APC function. In this paper we characterize the role played by Mf-derived EVs, their impact on antigen presenting cell function, and how this affects antigen-specific T-cell effector responses, particularly those responses driven by viral antigens. Our data suggest that EVs are central drivers of diminished cytokine production from APCs following activation that in turn downregulates antigen-driven T cell effector function.

## 2 Methods

### 2.1 Preparation of microfilariae, E/S products and EVs

Live *B. malayi* Mf were provided by the University of Georgia, Athens, GA by peritoneal lavage of infected jirds and were purified as previously described (8). To prepare Mf E/S products (supernatant), live Mf were thoroughly washed with culture media (RPMI 1640, 1% D-glucose, 1% L-glutamine, 1% penicillin/streptomycin) and maintained in culture media at a concentration of 1x10^6^ Mf/mL at 37°C for 24h. Mf were then pelleted, and the supernatant was collected. EVs were collected as previously described (17). Briefly, the ExoQuick-TC ULTRA kit (System Biosciences, Palo Alto, CA) was used, according to the manufacturer’s instructions, to isolate Mf-derived EVs after 24 hours of incubation. To perform the depletion of EVs from Mf E/S, E/S was collected and subjected to ultracentrifugation at 100,000 X g for 1 hour. The EV-depleted supernatant was collected and the pelleted EVs were discarded.

### 2.2 Cell culture

Human monocytes used for this study were isolated from leukopaks from healthy donors by counterflow centrifugal elutriation. This work was performed under Institutional Review Board (IRB)-approved protocols (99-CC-0168) from the Department of Transfusion Medicine (Clinical Center, National Institutes of Health, Bethesda, MD. All donors provided informed written consent. Human monocyte cells were isolated from 4 donors using magnetic beads (Pan Monocyte Isolation Kit, human, Miltenyi). 12x10^6 human monocyte cells were cultured for 6 days with addition of GMCSF and IL-4 to differentiate monocyte-derived DC.

Human DC were resuspended in complete culture media [RPMI 1640 medium (Gibco) supplemented with 10% heat-inactivated AB serum (Pan Biotech), 1% nonessential amino acids, 1% HEPES, 1 mM sodium pyruvate, 2 mM fresh L-glutamine, 100 μg/ml streptomycin, 100 units/ml penicillin (all from Life Technologies, USA)] and cultured in 6-well tissue culture plates at a concentration of 1x10^6^/mL. DC were activated with a final concentration of 100ng/mL IFN-y and 100ng/mL of LPS or incubated in media alone overnight. Lastly, DC were either left unconditioned (media), or conditioned with 50,000 live Mf, Mf-derived EVs (normalized using 10ug of protein content), 10uL of Mf E/S, or 10uL of Mf supernatant depleted of EVs for 48hrs at 37°C in 5% CO2.

Due to inherent limitations in sample availability and experimental design constraints, donor cells and cell lines were not uniformly accessible across all analyses. RNA sequencing experiments were conducted on a subset of 4 donors, while cytokine profiling encompassed 6 donors, with an additional cohort of 6 donors utilized for supplementary extracellular vesicle-dependent cytokine analyses. In a related investigation exploring T cell lineage dynamics in the context of COVID-19, a more extensive donor pool of 11 individuals was employed, as elucidated in the referenced study (19).

### 2.3 Cytokine and chemokine measurements

Supernatant from DC cultures was collected and assessed using a customized assay (Millipore HCYTOMAG-60K) for 10 human analytes: IL-10, IL-1a, IL-1b, IL-8, IL-12p70, RANTES, TNF-α, and IL-12p40. The assay was performed according to the manufacturer’s instructions, and samples were analyzed using a Bio-Plex 200 (Bio-Rad).

### 2.4 Preparation and analysis of samples for mRNA-Seq

RNA was extracted from 1x10^6 DC exposed to media (unconditioned), Mf, EV, E/S and EV-depleted E/S, from 4 different donors, using the Total RNA and Protein Isolation Kit (Thermo Fisher Scientific) following the manufacturer’s instructions. 1ul of total RNA from each sample was used to assess its concentration using a Qubit 4 (Invitrogen). 2ul of a normalized concentration of RNA from each sample was loaded into an Agilent RNA 6000 Nano LabChip using manufacturer recommendations and assessed through an Agilent 2100 bioanalyzer. The Agilent 2100 bioanalyzer calculated and reported RNA Integrity Numbers (ratio of 28S:18S ribosomal RNA) to determine total RNA sample quality. Next, mRNA was purified from total RNA using the RNeasy Pure mRNA Bead Kit (Qiagen) according to manufacturer instructions. Sample concentrations were normalized identically to total RNA normalization. 1ul of a normalized concentration of mRNA from each sample was loaded into an Agilent RNA 6000 Pico LabChip (Agilent Technologies) using manufacturer recommendations and assessed through an Agilent 2100 bioanalyzer. Samples with RIN values above 8.0 were prepared for mRNA-Seq analysis.

Bulk mRNA-Seq was performed by the Frederick National Laboratory for Cancer Research Sequencing Facility. Indexed RNA sequencing (RNA-seq) libraries were constructed from 1 µg total RNA using a TruSeq Stranded mRNA Library Prep (Illumina). 20 pooled mRNA-Seq samples were sequenced in paired-end mode using 1 lane of Illumina HiSeq3000/4000 flowcell, generating 2 × 151 bp reads. The mapping statistics are calculated using Picard software. Basecalling was performed using RTA (v.2.11.3). Samples were demultiplexed using Bcl2fastq v217) and aligned using STAR 2.7.0f. Library complexity is measured by unique fragments in the mapped reads using Picard’s MarkDuplicate utility.

### 2.5 Sequencing data analysis

The mRNA sequencing data was processed using default parameter with RNA-seek workflow (https://github.com/OpenOmics/RNA-seek). This included preprocessing of the raw sequencing reads to remove low quality bases, adapters, and shorter reads using Cutadapt v1.18, followed by alignment to human hg38 reference genome (GRCh38, Gencode Release 30) using STAR (v.2.7.6a) aligner in two-pass mapping mode. The gene expression counts estimated from number of reads mapping to each annotated gene using RSEM (v.1.3.0). The expression data were transformed to log2-counts per million (logCPM) and then filtered to remove genes with <1 CPM that are expressed in less than 4 samples. The filtered gene expression count was normalized using TMM (Trimmed Mean of M-values) method (20). Normalized data was then used for differential expression analyses using limma. The variation in gene expression data originating from cell-line donor patients was blocked for this analysis. EV-Media differential expression genes (FDR=0.05) were assessed using canonical pathway enrichment profiles generated using Ingenuity Pathway Analysis (IPA, Qiagen, Redwood City, CA, US). Information on these files may be found in an online repository (GEO: GSE263690 and GEE263693- see **Supplementary Tables 1**, **2** for associated metadata).

### 2.6 RNA isolation for RT-PCR

10x10^6^ monocyte-derived DC were cultured in complete media and exposed to purified EVs for 48hrs. RNA was extracted from EV-conditioned and unconditioned (media) DC using the Total RNA and Protein Isolation Kit (Thermo Fisher Scientific) following the manufacturer’s instructions. Using the Taqman Advanced RNA cDNA Synthesis Kit (Thermo Fisher Scientific) cDNA was made. 1–10 ng of RNA extracted from cells was used to perform polyadenylation tailing reaction at 37°C for 45 minutes and then 65°C for 15 minutes. This was followed by adenylation ligation reaction that was performed at 16°C for 60 minutes. The reverse transcriptase reaction was performed at 42°C for 15 minutes followed by 85°C for 5 minutes. An amplification step was performed at 95°C for 5 minutes for 1 cycle, 95°C for 3 seconds for 14 cycles, 60°C for 30 seconds for 14 cycles, and 99°C for 10 minutes for 1 cycle. RT-PCR was performed to detect mRNA expression of genes identified within the RNASeq analysis. The reaction mix contained 18ul of cDNA, 2 ul of each respective primer and 20ul of Taqman Advanced Mastermix. Cycling parameters were 95°C for 20 seconds for 1 cycle, 95°C for 40 cycles and 60°C for 40 cycles. A Ct value of 40 is negative. All of the primers used were purchased commercially from Bio-Rad: (PrimePCR™ SYBR^®^ Green Assay: TNC, Human (#10025636), PrimePCR™ SYBR^®^ Green Assay: MT1F, Human (#10025636), PrimePCRSYBR^®^ Green Assay: SLC7A11, Human (#10025636), PrimePCR™ SYBR^®^ Green Assay: PAPPA2, Human (#10025636), PrimePCR™ SYBR^®^ Green Assay: JAK3, Human (#10025636), PrimePCR™ SYBR^®^ Green Assay: SLC39A10, Human (#10025636), PrimePCR™ SYBR^®^ Green Assay: TLR7, Human (#10025636), PrimePCR™ SYBR^®^ Green Assay: S100A4, Human (#10025636), PrimePCR™ SYBR^®^ Green Assay: SLAMF1, Human (#10025636), PrimePCR™ SYBR^®^ Green Assay: mTOR, Human (#10025636)).

### 2.7 RT-PCR data handling

The 2^DeltaDelta (Ct) formula was used to calculate fold change in gene expression. The average Ct of EV-treated DC, untreated (media) DC, and 18s (housekeeping gene) were calculated for each gene. Average 18s Ct values were subtracted from all genes to calculate the Delta Ct values for EV-treated DC and untreated DC replicates. Next, the Media delta Ct values were subtracted from the EV-treated delta Ct values to calculate delta delta Ct values. Lastly, to calculate fold change, 2^ (delta delta Ct values) were calculated for EV-treated cells.

### 2.8 Conditions for filarial helminth exposed DC and SARS CoV-2-membrane specific T- cell lines

As previously described, we generated SARS-CoV-2 Membrane protein-specific CD4+ T cell lines (TCLs) from 6 donors (19) who provided PBMCs under a NIAID IRB approved protocol (88-I-0083) (19). For *in vitro* expansion of these CD4^+^ TCLs, cryopreserved Membrane specific-TCLs were thawed and incubated for 12 days in the presence of the respective antigens, irradiated autologous feeder PBMCs, and 60U of rIL-2, as previously described (REF). Separately, autologous monocytes were purified from cryopreserved PBMCs from whom the TCLs were generated using negative depletion magnetic cell sorting (MACS Miltenyi Biotec, USA). 12x10^6 purified monocytes were seeded in 6-well plates and placed in R10 media culture [RPMI 1640 medium (Gibco) supplemented with 10% heat-inactivated AB serum (Pan Biotech), 1% nonessential amino acids, 1% Hepes 1M, 1 mM sodium pyruvate, 2 mM fresh L-glutamine, 100 μg/ml streptomycin, 100 units/ml penicillin (all from Life Technologies, USA)] at 37°C, 5% CO2 for 6 days to be differentiated into DC with addition of 10uL of IL-4 (1mg/mL) and 10uL of GM-CSF (1mg/mL) at days 1,3, and 5. 1x10^5 DC were seeded into 6-well plates and exposed to 50,000 live Mf, EVs or media alone for 48hours in 37C with 5% CO2. After treatment conditioning, DC were washed and loaded for 4hrs with 1µg/mL of SARS-CoV-2 peptide megapools (MPs).

Next, all DC conditions were counted and seeded in 96-well round bottom culture plates by condition (Mf-conditioned, EV-conditioned, media/unconditioned). SARS-CoV-2 Membrane-specific T-cells from autologous donors were added in a ratio of 1 DC:10 CD4+ T-cells in each plate. DC: TCL co-cultures were unstimulated (media) or stimulated with 1ug/mL Membrane protein, or 1ug/mL SEB for 17hrs in 37C. BFA was added after 6hrs of stimulation.

### 2.9 Flow cytometry conditions for filarial helminth exposed DC and CD4+ SARS CoV-2- membrane specific T- cell lines

The activation and intracellular cytokine profiles of the SARS-CoV-2-Membrane specific CD4+ TCLs incubated with their autologous Mf and EV-conditioned DC were analyzed using flow cytometry immunophenotypic and functional assays. Briefly, TCLs were stimulated overnight in 5% CO2 at 37°C with autologous donor SARS-CoV-2 antigen-loaded monocyte-derived DC conditioned with Mf and EV or unconditioned (media). Cells were stained for viability (Live/Dead fixable blue, UV450, Molecular Probes) and then incubated with anti-CD3 (BUV805, UCHT1, BD), anti-CD4 (cFluor YG584, SK3, BD) for 30 min in the dark at room temperature. The cells were then washed twice with FACS buffer and fixed using a Fix/Perm buffer kit (BioLegend) for 30 min at 4°C. The cells were washed twice with Perm buffer (BioLegend) and resuspended with the intracellular antibody mix containing anti-CD69 (BV711, FN50, Biolegend), anti-CD154 (PE, TRAP-1, BD), anti-IFN-γ (PE-Cy7, B27, BD) for 30 min at 4°C. Finally, the cells were washed twice with Perm buffer and acquired using the Cytek Aurora flow cytometer (Cytek Bio) and Spectroflo software (Cytek Bio) for acquisition. FCS files were analyzed using OMIQ software (Dotmatics) and cell frequencies and ratios were exported to GraphPad Prism 7 for analysis.

### 2.10 Statistical analysis

For Luminex assay and flow cytometry cell frequencies/MfI results, paired samples were analyzed using the Wilcoxon test, and the Mann-Whitney test (nonparametric test) was used to compare among different groups. The geometric mean was used as a measure of central tendency. GraphPad Prism 7 was used for all statistical analysis. *P* values less than.05 were considered significant.

## 3 Results

### 3.1 Microfilaria-derived EVs downregulate activated DC cytokine production

**Figure 1:**
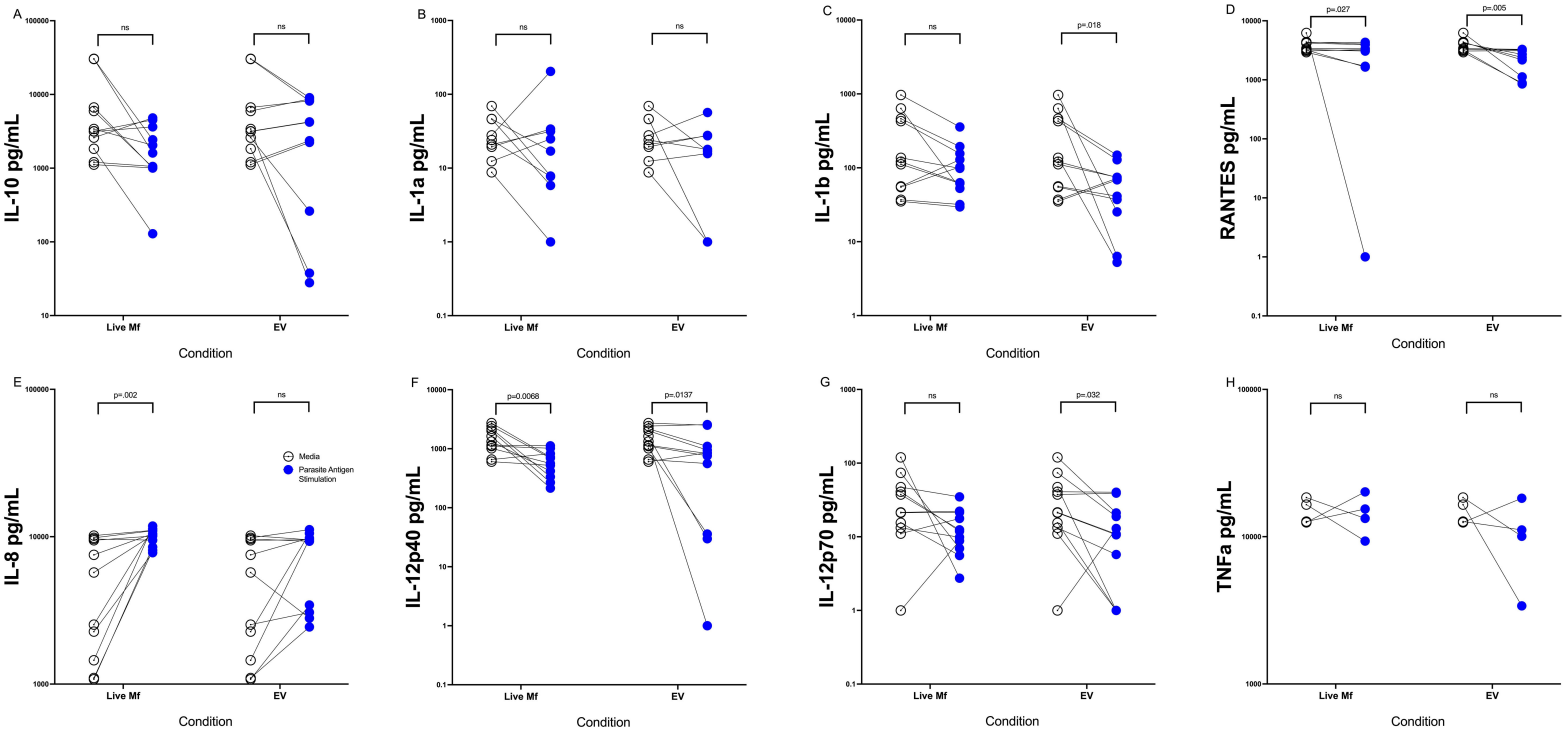
EVs downregulate cytokine production in activated DC Human DC (n=10-11) after exposure to media alone (open circles), live Mf (closed circles) or EV (closed circles) were activated with LPS and IFN-y and supernatants assessed for production of IL-10 **(A)**, IL-1a **(B)**, IL-1b **(C)**, RANTES **(D)**, IL-8 **(E)**, IL-12p40) **(F)**, IL-12p70 **(G)**, TNF-α **(H)**. NS, non-significant.

Having previously shown that live Mf and Mf-derived E/S products alter APC function [34 (21)] EVs may downregulate APC mTOR phosphorylation (8, 17), we chose initially to corroborate some of these data and to assess the role of Mf-derived EVs in altering DC cytokine production both before and following activation. To this end, DC derived from monocytes from 11 healthy donors were stimulated with live Mf or with EVs for 2 days. As has been shown previously (22), compared to their respective baseline levels, both live Mf and Mf-derived EVs were capable of inducing significantly higher amounts of (p<.05 for all cytokines) IL-10, IL-8, RANTES and TNF-α (**Supplementary Figure 1**) compared to unexposed DC. IL-12p40 and IL-12p70 were not induced following exposure to live Mf or Mf-derived EVs. We next assessed differences in cytokine induction by Mf- or EV-conditioned DC following activation with LPS and IFN-γ. As can be seen in **Figure 1**, IL-1b (p=.019), IL-12p40 (p=.014), RANTES (p=.005), and IL-12p70 (p=.032) levels were statistically significantly lower in EV-treated human DC than in those left unexposed; RANTES (p=.027) and IL-12p40 (p=.0068) induction was also statistically significantly lower in Mf-treated DC when compared to Mf-unexposed DC. There were no statistically significant differences seen between Mf- or EV-exposed IL-10, IL-1α or TNF-α production compared to unexposed DC. The percentage of inhibition of cytokine production was calculated by comparing the average cytokine pg/mL production per treatment condition (EV-conditioned DC vs. Media). All cytokines assessed, except for IL-8, demonstrated inhibition in cytokine production as a consequence of EV-conditioning: IL10 (52.85%), IL12p40 (60.58%), IL8 (-33.63%), 12p70 (40.52%), RANTES (56.54%), TNF (71.17%), IL1 (60.1%), and IL1b (22.23%). These data indicate EVs diminish DC cytokine production following activation to the same degree as live Mf.

### 3.2 EVs downregulate IL-10 and IL-12p40 cytokine induction in DC

**Figure 2:**
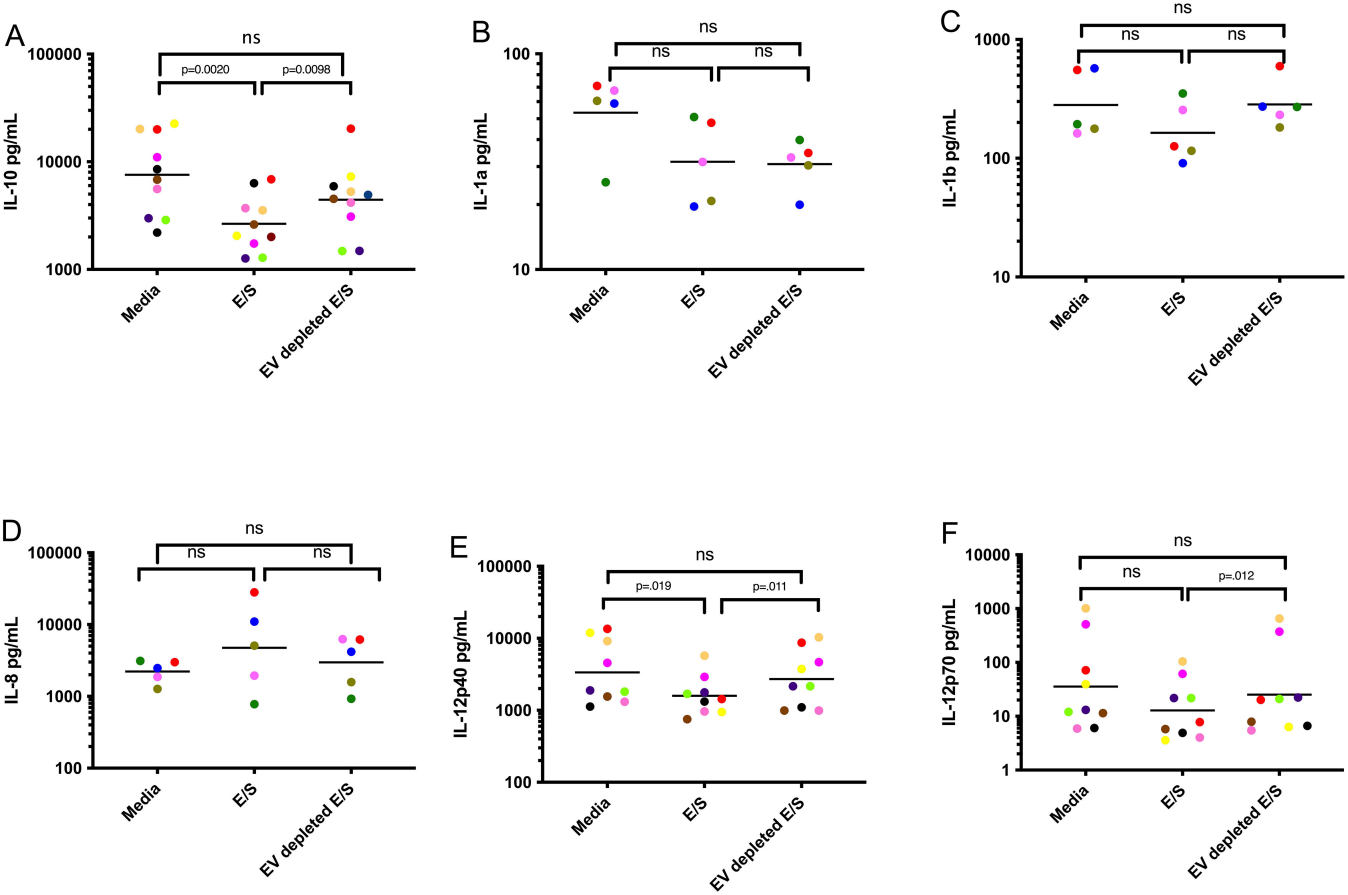
EVs contained within Mf E/S drive DC suppression of cytokine production following activation Human DC were exposed to media, Mf-derived E/S, and Mf-derived E/S depleted of EV for 48 hours and assessed for IL-10 **(A)**, IL1a **(B)**, IL1b **(C)**, IL8 **(D)**, IL-12p40 **(E)**, IL-12p70 **(F)**. Each dot represents an individual DC culture with the color matched for each donor among conditions. ns, non-significant.

Having found that EV exposure downregulates cytokine production from DC following activation, we next assessed the role of EVs in driving alterations in DC function. By comparing E/S and E/S depleted of EV (**Figure 2**), we could show that DC, when conditioned with E/S and activated with LPS/IFN-γ, markedly downregulated the production of IL-10 (**Figure 2A**) and IL-12p40 (**Figure 2E**) when compared to DC conditioned with media alone. When exposed to E/S depleted of EVs, the IL-10, IL-1a, IL-1b, IL-1c, IL-12p70 and IL-12p40 levels were no different than DC conditioned by media alone (p>.05 for all comparisons) following activation (**Figures 2A–F**). These data indicates that EVs contained within the E/S are likely responsible for the alteration of DC cytokine production following activation with LPS/IFN-γ.

### 3.3 EVs are central drivers of gene function alterations in human DC

**Figure 3:**
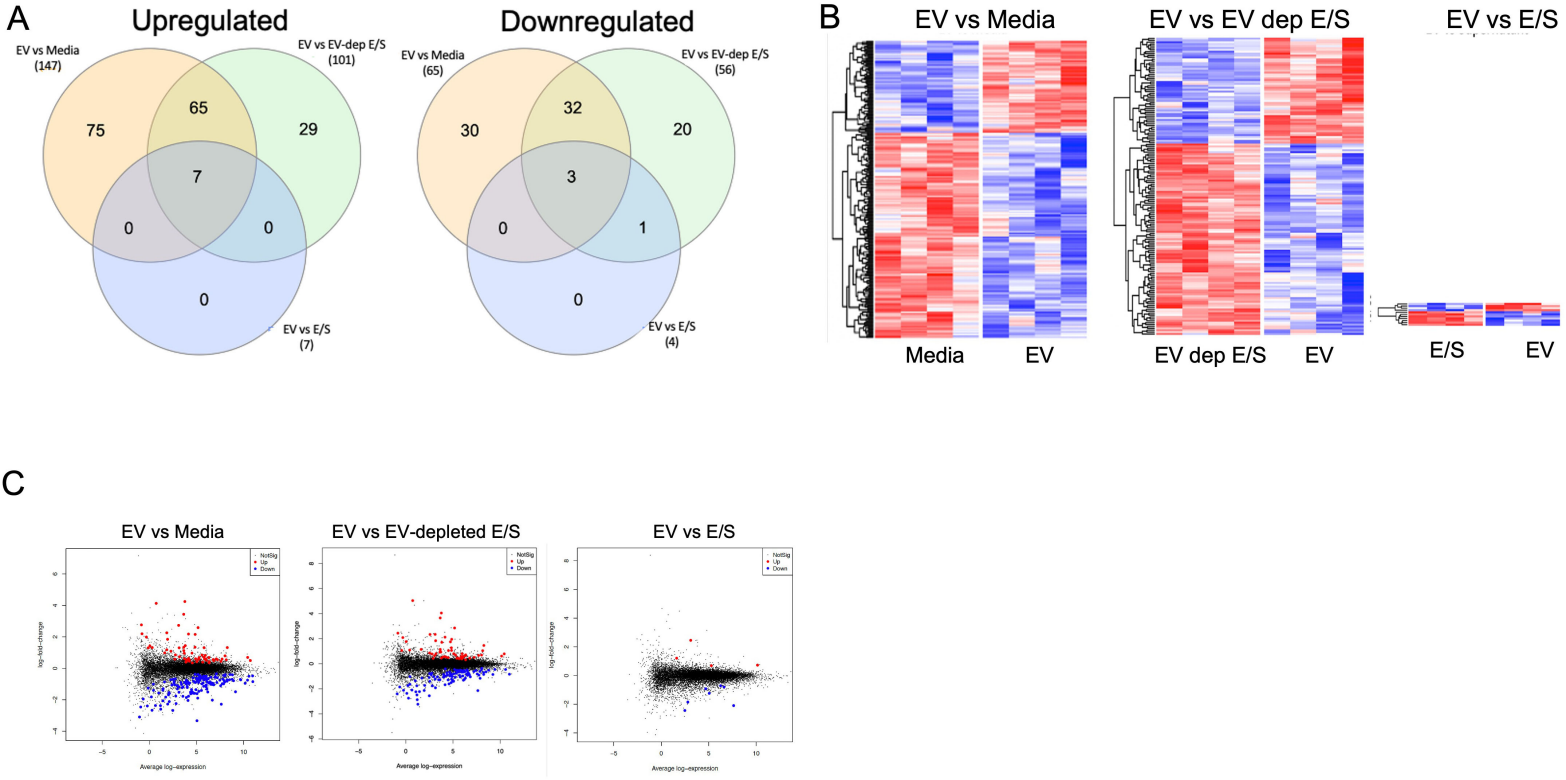
Differentially expressed genes (DEGs) in antigen presenting cells exposed to filarial helminth byproducts RNA sequencing results from 4 separate human DC donors left unexposed (media) or exposed to Live Mf, EVs, EV-depleted E/S, and E/S. **(A)** Shows two Venn diagrams of the number of upregulated (Left) and downregulated (Right) DEGs among the various conditions. **(B)** Shows the unsupervised hierarchal clustering of DEGs for EV vs Media (Left, heatmap), EV vs EV-depleted E/S (middle, heatmap), and EV vs E/S (right, heatmap). **(C)** Depicts volcano plots of the various comparisons with blue dots represents statistically significant downregulated DEG and the red dots representing significantly upregulated DEG.

Next, we assessed the impact of Mf-derived EV exposure on the function of DC through RNASeq analysis. Human DC were treated with Mf-derived EVs, E/S, or EV-depleted-E/S. Following exposure, cells were harvested, RNA prepared and sequenced. Using a false discovery rate (FDR)<0.05, we found EV-exposed DC had 212 differentially expressed genes (DEGs) when compared to EV-unexposed DC (147 upregulated/65 downregulated). Most interestingly, there was essentially no difference in DEGs between DC exposed to EVs and those exposed to E/S (only 10 [7 upregulated, 3 downregulated] DEGs. Like the comparison between EV-exposed and media-exposed DC, EV-depleted E/S looked similar to media when compared to EVs (157 DEGs [56 downregulated/101 upregulated (**Figure 3A**). Clustering of the DEGs across all conditions showing all 4 donor DC, demonstrate that EVs contained within the E/S were responsible for the effects observed in E/S products. Our findings highlight the central role of EVs derived from Mf in altering the gene expression and function of DC.

**Figure 4:**
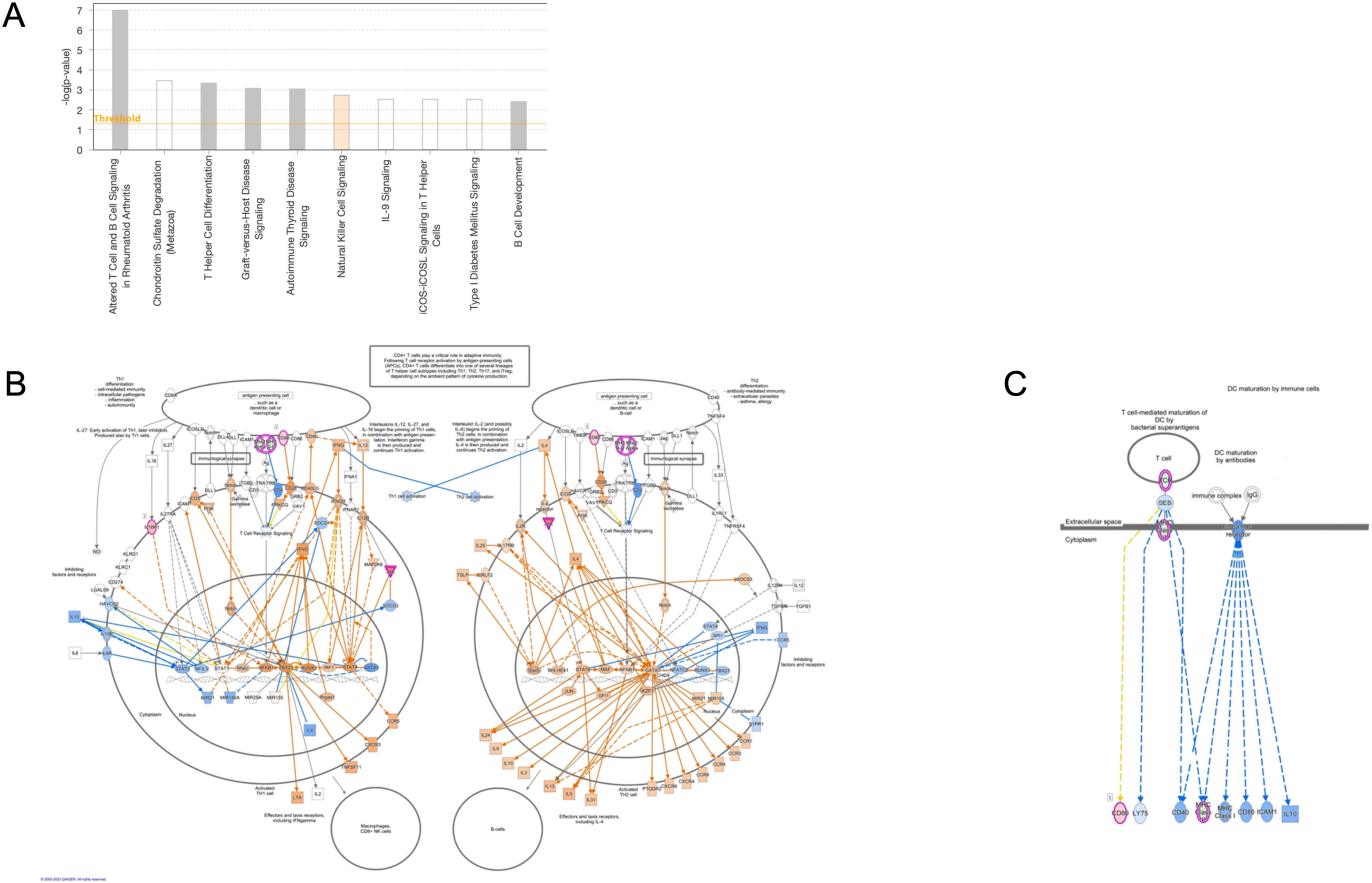
Ingenuity Pathway Analysis of annotated canonical pathways in EV-conditioned DC Top Canonical signaling pathways of biological relevance in human DC **(A)**. Map of the Th1 and Th2 activation pathway with blue indicating downregulated genes in the pathway **(B)**. A map of the DC Maturation canonical pathway indicate annotated molecules are associated with suppression (blue) of Fc gamma receptor signaling **(C)**.

Ingenuity Pathway Analysis (IPA) was used to uncover pathways in DC modulated by exposure to microfilariae (Mf)-derived EVs. The analysis identified two predominant enriched canonical pathways: “DC Maturation” and “Th1 and Th2 Activation Pathways” (**Figure 4A**). Within the IPA-identified DC Maturation canonical pathway, genes associated with antigen processing (HLA-DMA, HLA-DMB) and immune complex recognition (Fc-gamma receptor, FcγR) were found to be downregulated. The Fc-gamma receptor component encompassed annotations for inhibitory molecules and demonstrated downregulatory relationships between MHC Class I and II activation and IL-10 production. Notably, the MHC Class II genes HLA-DMA and HLA-DMB, crucial for antigen presentation, exhibited marked downregulation within this pathway. This downregulation aligns with the overall inhibitory pattern observed in the DC Maturation pathway, suggesting a potential mechanism by which Mf-derived EVs may modulate DC function and subsequent immune responses. These findings provide insights into the molecular mechanisms underlying the immunomodulatory effects of Mf-derived EVs on DC, potentially contributing to our understanding of host-parasite interactions in filarial infections.

Because APCs drive activation of T cells, it is not surprising that the second most important canonical pathway was that associated with Th1 and Th2 activation. Th1 and Th2 activation is pleiotropic with multiple molecules within the pathway responsible for elements of cellular proliferation regulation. As can be seen (**Figures 4A–C**), EV-treated cells demonstrate inhibition in Th2 cell proliferation indicated by a significantly downregulated, negative z score describing the pathway regulatory networks.

We next used real-time quantitative RT-PCR to confirm the differential gene expression found in the RNAseq analysis. DC were either exposed to EVs or media and RNA was prepared from these cells. Using genes identified to be relevant to both viral and helminth immune response, we were able to independently corroborate the EV-induced alteration of M1 (Signaling Lymphocytic Activation Molecule Family Member 1), TLR7 (Toll-Like Receptor 7), SLC (Solute Carrier Family 7 Member 11), MT1 (Metallothionein 1F) and mTOR (Mechanistic Target of Rapamycin Kinase) (**Supplementary Figure 3**).

### 3.4 Mf-derived EVs alter DC activation and activity of antigen-specific T-cells

**Figure 5:**
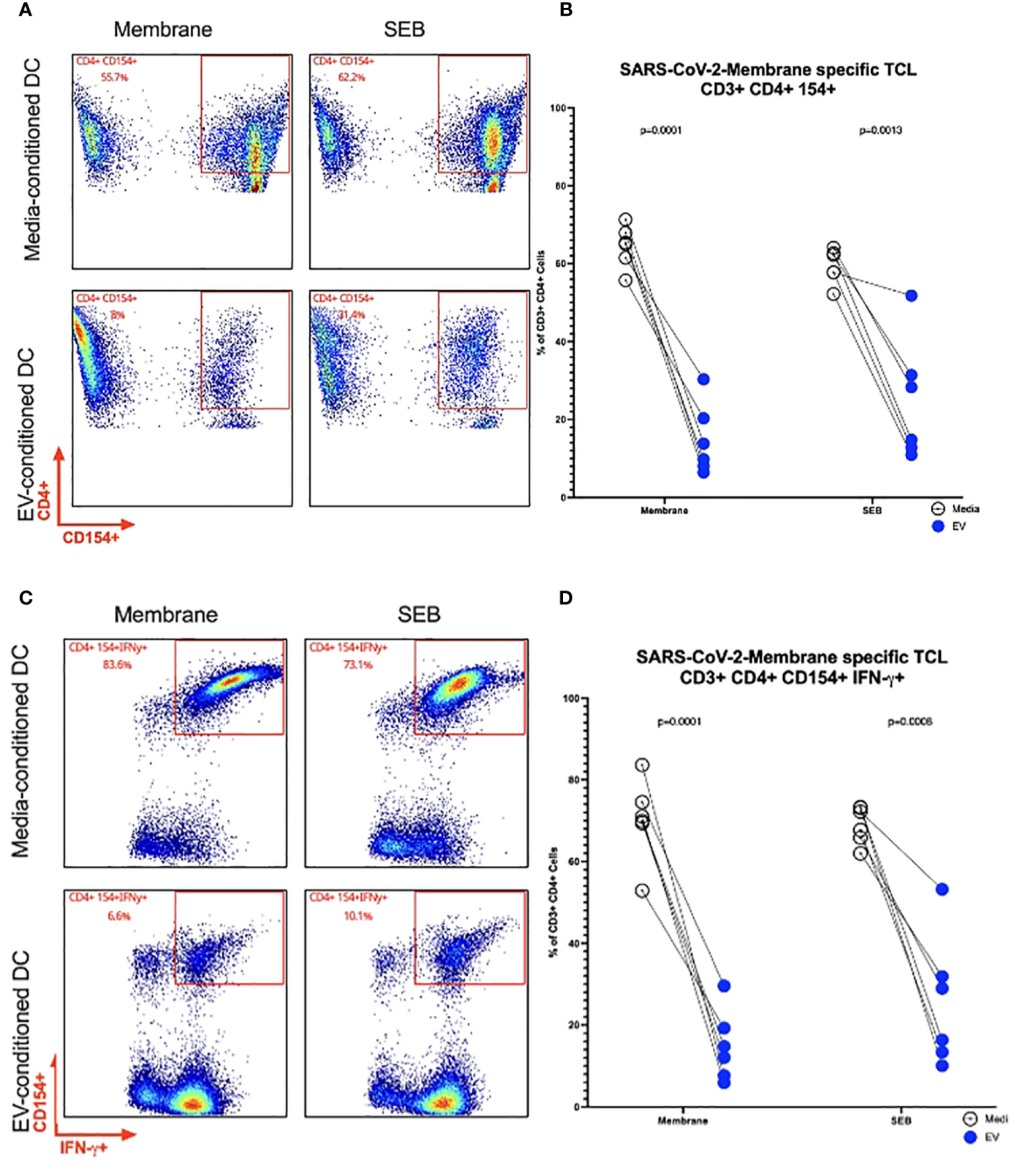
Frequency of SARS CoV-2 Membrane TCLs following activation by EV-conditioned human DC Representative flow cytometry plots of a single patient for SARS-CoV-2 Membrane-specific CD3+ CD4+CD154+ T cells **(A)**. Frequencies of SARS-CoV-2 Membrane-specific CD3+ CD4+CD154+ T cells **(B)**. Representative flow cytometry plots of a single patient for SARS-CoV-2 Membrane-specific CD3+ CD4+ CD154+ IFN-y+ **(C)**. Frequencies of SARS-CoV-2 Membrane-specific CD3+ CD4+CD154+ T cells detected in coincubations with EV-conditioned dendritic cell **(D)** n= 6.

To determine the impact of Mf-derived EVs as modifiers of DC function, we investigated the impact of EVs on DC activation of antigen-specific CD4+ TCLs (19). Using autologous DC derived from the 6 donors from whom the TCLs were derived, these DC were exposed to Mf-derived EVs or media for 48hrs. After 48hrs, the cells were washed and loaded with SARS CoV-2 Membrane peptide pools. Concurrently, Membrane-specific (n=6) TCLs were thawed and co-incubated with their matched (and conditioned) DC. Flow cytometry to assess intracellular CD154, a marker of antigen-experienced CD4 T cells, and IFN-γ, a marker of an anti-viral cytokine response were assessed (**Supplementary Figure 3**). As can be seen in **Figure 5**, using SARS CoV-2 Membrane-specific CD4+ T cell derived from the same 6 donors and DC conditioned with Mf-derived EVs, we were able to find fewer activated SARS CoV-2 Membrane-specific CD3+ CD4+ CD154+ cells (p=.0001) when compared to unconditioned DC. Additionally, Mf-derived EV-conditioning of DC induced fewer SARS CoV-2 Membrane-specific CD3+ CD4+ cells capable of producing IFN-γ (p=.0001).

## 4 Discussion

*Brugia malayi* represent a class of parasitic (filarial) organisms that inhabit the lymphatic system and can induce subclinical chronic infections in their hosts. The disease associated with infection (lymphatic filariasis) can be characterized by significant morbidity resulting from the inflammatory responses elicited by the filarial worms. Recent studies have revealed that filarial helminths secrete EVs containing a variety of biomolecules, including small RNAs, proteins, lipids, and glycans, which are then internalized by host cells (13, 17). Our research group has previously demonstrated that both the Mf and the EVs derived from them are capable of modulating host mTOR-associated pathways which play critical roles in the regulation of cellular proliferation, autophagy, and apoptosis. The unique characteristics of EVs, including their ability to transport immunomodulatory cargo, have made them the focus of increasing attention as a potential therapeutic tool for targeted delivery of these biomolecules. Our investigations have shown that EVs are readily internalized by human DC, where they can modulate key intercellular functions (17). 21 proteins unique to *Loa loa* EVs have been found in patient sera, indicating potential use of EVs as a biomarker of infection (23). Additionally, the immunomodulatory functions of EVs have been explored as a potential therapeutic approach for reducing clinical symptoms and inflammatory markers in models of inflammation (24). In particular, studies of the effects of helminth infections on immune responses to viral antigen and vaccines have revealed that these infections induce a cytokine response that diminishes bystander antigen-specific immune responses (25). In the present study, we aimed to characterize the role of EVs in mediating alterations of human APC functions and to evaluate the immunomodulatory effects of these EVs on viral-specific immune responses.

EVs contain cargo that modulate the functions of DC and influence their interactions with immune cells. Through RNA sequencing analysis, we demonstrated that EVs are a potent modulator of DC gene expression, as the gene expression in EV-exposed cells was found to be distinct from both EV-depleted E/S- and media-exposed cells (**Figures 3A-C**). These findings provide strong evidence that EVs, rather than unencapsulated soluble mediators, are responsible for driving the observed changes in DC functions. Our previous research has established that EVs, like Mfs can alter immune activation functions in APCs through their internalization within the cell cytosol; in so doing they appear to suppress the production of proinflammatory cytokines (26). In line with these findings, in the present study, exposure to Mf-derived EVs resulted in a suppression of DC cytokine production, including IL-12p40, IL-12p70, IL-1b, and RANTES in activated human DC (**Figures 1A, B**). Additionally, depletion of EVs from Mf E/S resulted in a return of these cytokines to their baseline levels. The impact of EVs on reducing cytokine secretion demonstrate their immunomodulatory capacity. The results presented in this study underscore the potential utility of EVs as a therapeutic tool, owing to their ability to internalize within a diverse range of target cells and transfer molecules that modulate intracellular machinery. Specifically, the findings of this study implicate EVs as important regulators of host-parasite immune responses and suggest that they have great promise as a means of manipulating immune activation and suppression. A deeper understanding of the underlying mechanisms by which EVs modulate these processes could inform the development of novel therapeutic strategies for the treatment of helminth infections.

In addition to their potential application in the context of parasitic infections, the results of this study further support the growing body of literature that indicates that EVs are key players in the regulation of APC function and, thus, are capable of shaping the host immune response (13, 27–29). We found EVs downregulate pathways for DC maturation that in turn have consequences for Th1 differentiation. The impact of reduced Th1-associated effector function include limitations to expansion of immune subsets, and dysregulated immune effector functions for antiviral responses.

We found that EVs downregulated MHC-II expression in DC as well as HLA-DMA and HLA-DMB, a marker of DC maturation. The enzyme HLA-DM facilitates CLIP dissociation from MHC-II, which enables the binding of a peptide to MHC-II. DM stabilizes MHC-II during peptide exchange and selects for peptides with higher binding affinities. Therefore, HLA-DM may play a role in DC antigen presentation by facilitating the exchange of peptides bound to MHC-II. EVs may alter HLA-DMA and HLA-DMB, preventing antigen-processing of filarial antigens to avoid immune detection.

As stated above, HLA-DM is a nonclassical MHCII-like protein that plays a pivotal role in selecting high specificity epitopes, ultimately shaping the adaptive immune response. By acting as a peptide exchange catalyst, HLA-DM helps to shape the MHCII immunopeptidome by editing the repertoire of presented peptides (30). The interaction between T-cell receptor (TCR), peptide and major histocompatibility complex (MHC) can determine Th1/Th2 dominance and selection of CD4+ T cell functions (31). In other parasitic infections, HLA-DM genes are also downregulated (32), suggesting EVs may inhibit high MHCII density on DC and alter Th1/Th2 differentiation (31). We also found that EVs altered the expression of FcγRs, receptors found on many myeloid cells that recognize targets coated with IgG, such as opsonized pathogens or immune complexes (33). Plasma from *Brugia* spp. Mf+ infected individuals actively impair granulocyte activation and degranulation, a process governed by FcgR-signalling, suggesting a possible role for EVs in altering the functionality of FcγRs (34).

Patent lymphatic filariasis, a chronic infection caused by B*. malayi* and the related parasite, *Wuchereria bancrofti*, is also characterized by defects in DC function and antigen-specific T-cell unresponsiveness (35). Previous research has shown that Mf exposure can reduce the expression of innate antiviral immune ligand receptors, such as TLR3 and TLR4 which can lead to a diminution of antigen-specific responses (36).

These alterations in the immune environment can have long-lasting consequences for vaccine and viral bystander memory. Our current study found that virus (SARS CoV-2 Membrane)-specific TCLs had lower frequencies of activated cells and fewer IFN-γ producing cells when cultured with EV-conditioned DC (**Figure 5**) This suppression of T-cell activation is consistent with the findings of previous studies, which have shown that chronic filarial infections are associated with lower levels of inflammatory cytokines such as IFN-γ, which is partially due to T-cell exhaustion. Helminth infections, including *B. malayi* infections, produce exhausted T cells with increased expression of inhibitory receptors (PD-1, LAG3, CTLA4) that are capable of downregulating T-cell proliferative responses and increasing the potential for apoptosis (2, 37–39).

In conclusion, our study provides further evidence of the potential of EVs as downmodulatory of host immune responses. These findings suggest that many of the modulatory effects seen in chronic filarial infection are mediated through the internalization of EVs on APCs. Further research will be needed to fully characterize the fine intracellular details of EV trafficking and immune modulation, as well as to investigate the therapeutic implications of these.

## Data Availability

The data presented in the study are deposited in the GEO repository and is publicly available under accession number GSE263690.
