# Supplementary material for: Brugia malayi filarial helminth-derived extracellular vesicles suppress antigen presenting cell function and antigen-specific CD4+ T cell responses
Source: Front Immunol. 2024 Oct 7;15:1436818. doi: 10.3389/fimmu.2024.1436818 (PMC11491353; doi:10.3389/fimmu.2024.1436818)
Supplement: Supplementary file 1 [file DataSheet1.docx]

Supplementary Material

# Supplementary Data

# Supplementary Figures and Tables

## Supplementary Figures


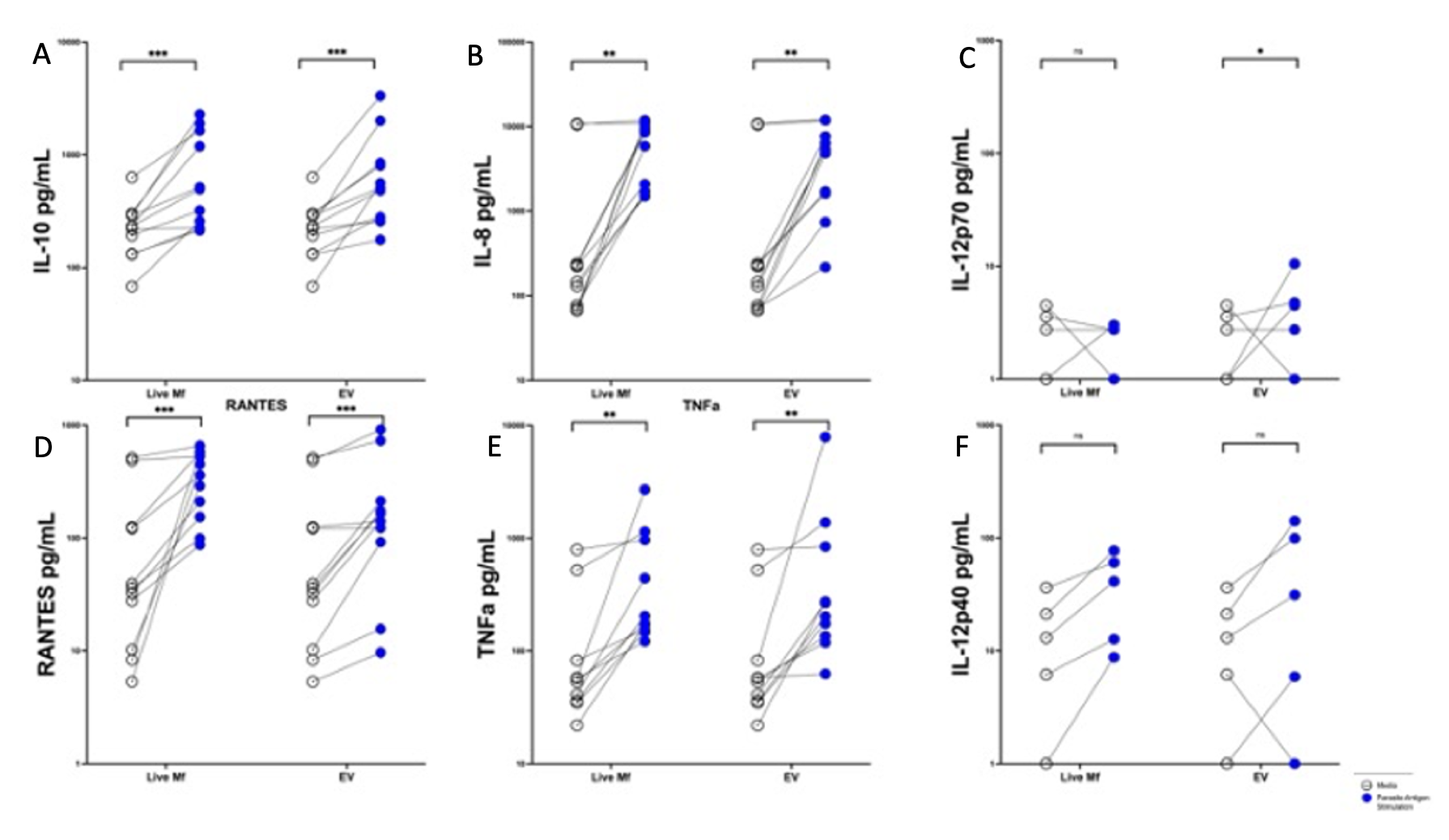


**Figure S1: Cytokine production in dendritic cells after co-incubation with Mf and EVs** Monocyte-derived dendritic cells were placed in culture alone(media), with live Mf, or EV for and cell supernatant was collected from each condition and assessed for cytokine production of IL-10(n= 11)(Panel A), IL-8(n=11)(Panel B), IL-12p70(n=5)(panel C), RANTES(n=11)(Panel D), TNF-α(n=10)(Panel E), IL-12p40(n=5)(Panel F).

**Figure S2:** **Dendritic Cell conditioning and presentation to SARS CoV-2 Spike and Membrane-specific TCLs** Autologous monocytes were differentiated into DCs and exposed to live Mf, EVs or media alone. Following conditioning with filarial helminths and EVs, DCs were loaded with SARS-CoV-2 peptide megapools (MPs) consisting of SARS-CoV-2 Spike protein (MP-S) and SARS-CoV-2 Membrane protein (MP-M) and co-cultured with autologous SARS-CoV-2-Spike and SARS-CoV-2 Membrane-specific T-cells for 17 hours with or without stimulation and assessed by flow cytometry using a Cytek Aurora platform.


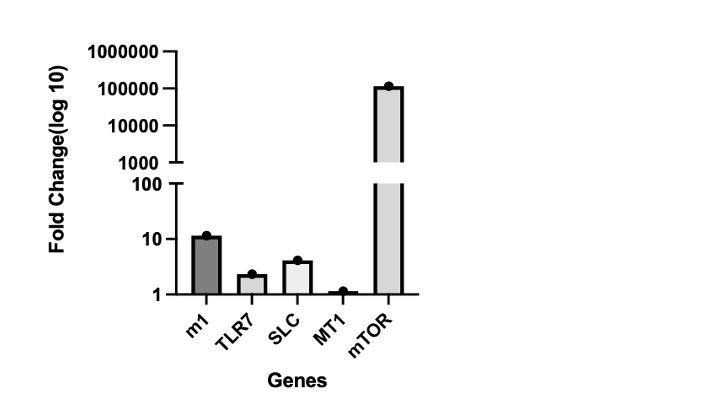


**Figure S3:** **Mf-derived EVs downregulate dendritic cell M1, TLR7, SLC, MT1 and mTOR gene function** RNA was isolated from 10x10^6 cells and converted into cDNA for analysis by RT-PCR . EVs downregulate mRNA expression of M1, TLR7, SLC, MT1 and mTOR. EV-conditioned monocyte mRNA expression shown as fold change from unexposed monocytes using real-time RT-PCR relative to ribosomal RNA

## Tables

Table S1: Metadata related to human monocytes following exposure to Brugia malayi filarial helminth-derived extracellular vesicles (GEO: GEE263693)

Table S2: Metadata related to human dendritic cells following exposure to live Brugia malayi filarial helminth, EVs, E/S, or EV-depleted E/S (GEO: GEE263690).
